# Effects of the School-Based Integrated Health Promotion Program With Hydroponic Planting on Green Space Use and Satisfaction, Dietary Habits, and Mental Health in Early Adolescent Students: A Feasibility Quasi-Experiment

**DOI:** 10.3389/fpubh.2021.740102

**Published:** 2021-09-24

**Authors:** Stephen Wai Hang Kwok, Cynthia S. T. Wu, Hiu Tung Tong, Chun Ni Ho, Ka Lee Leung, Yolanda C. P. Leung, Kam Chung Lui, Carson K. C. Wong

**Affiliations:** School of Nursing, The Hong Kong Polytechnic University, Kowloon, Hong Kong, SAR China

**Keywords:** school-based, integrated health promotion, hydroponic planting, green space, dietary habits, mental health, adolescent students

## Abstract

**Background:** School-based green space activities have been found to be beneficial to the physical activity level and lifestyle habits of adolescent students. However, their effects on green space use and satisfaction, mental health, and dietary behaviors required further investigation. This study aimed to investigate the effects of school-based hydroponic planting integrated with health promotion activities in improving green space use, competence and satisfaction, healthy lifestyle, mental health, and health-related quality of life (QoL) among early adolescent students in secondary schools.

**Methods:** This study adopted a three-group comparison design (one control and two intervention groups). Secondary school students (*N* = 553) of grades 7–9 participated in either (1) hydroponic planting (two times per week for 8 months) integrated with health promotion activities; (2) only health promotion activities (one time per week for 6 weeks); or (3) control group. Outcomes assessed by questionnaire included green space use and satisfaction, life happiness, lifestyle, depressive symptoms, and health-related QoL.

**Results:** After adjusting for sex and school grade, the scores in “green space distance and use” and “green space activity and competence” were significantly better in the intervention groups than in the control group. Hydroponic planting integrated with health promotion activities was also associated with better scores in dietary habits and resistance to substance use. Intervention groups had a higher score in “Green space sense and satisfaction” and life happiness when compared with the control group.

**Conclusions:** Our study shows that the school-based hydroponic planting integrated with health promotion activities were feasible and, to a certain extent, useful to improve green space use and competence, dietary habits, and resistance to substance use among early adolescent students in secondary schools in urban areas. Future studies should address the limitations identified, for example, designing a randomized controlled trial that could fit school schedules to generate new evidence for physical and mental health in adolescent communities.

## Introduction

Early adolescents have been exposing to several risk factors for physical and mental well-being in Hong Kong and similar cities in developed regions. School-based health intervention needs to be developed and implemented focusing on environmental factors, lifestyle, mental health, and related constructs. For example, less than half of the junior secondary school students in Hong Kong had breakfast everyday; their consumptions of fruits and vegetables were lower than the daily intake recommended by the Hong Kong Department of Health (1). Similarly, in the United States, the recommendations for eating fruits and vegetables were infrequently followed among adolescents (2). A diet low in fruits was associated with a higher body mass index (BMI) (3). On the other hand, a diet high in fruits and vegetables was associated with a lower risk of chronic diseases. WHO (4) identified that unhealthy diets, among other risky lifestyles, were risk factors for major non-communicable diseases (NCDs). Although several factors, such as income, knowledge, personal preferences, and environmental factors, could affect dietary habits, WHO recommended that measures, standards, and policies should be put in place to ensure affordability, availability, accessibility, and acceptability of fruits and vegetables in school communities.

Furthermore, mental health issues were found to be linked to dietary behaviors. Adolescent students, to a certain extent, have been facing stress from different sources such as academic stressors or family issues. A previous study found that stress-driven eaters had a higher prevalence rate of being overweight and obese; and the stress-related eating behavior was more common in female adolescents (5). Hong Kong students are at risk of stress-associated eating since academic stressor is a main source of stress, which could lead to mental health issues (6). WHO (7) reported that around half of all mental health disorders in adulthood start by age 14, and mental health conditions accounted for 16% of the global burden of disease and injury in adolescents. The population aged between 10 and 14 belong to the stage of early adolescents, who should be educated about healthy lifestyle (8). Compared with modifying health-related behaviors in adulthood, the changes could be easier during childhood (9). Healthier students are usually better learners (10) and have greater academic achievement and higher graduation rates, which would further translate into lifelong benefits (11).

Therefore, school is the ideal place for health education (8). Evidence from randomized controlled trials (RCTs) showed that school-based health promotion activities could improve the knowledge, attitudes, and dietary habits of students. A German study implemented a school-based health promotion program on over 1,900 school students in a state to observe changes in their dietary habits over 1 year. Students in the control group were found skipping breakfast more often than the intervention group in a cluster RCT design (12). Another RCT showed that school gardening interventions could have positive effects on fruit intake, trying new fruits, and recognizing vegetables among students (13). Further evidence supported the positive effects of school gardening on the dietary behaviors of students. Students who had undertaken nutrition education plus gardening exhibited improvements in relevant knowledge and taste appraisal as well as choosing and consuming vegetables at lunch compared with the control group (14). Previous studies also reported that school-based gardening increased daily consumption of fruits and vegetables, and increased intentions, attitudes, norms, and perceived behavioral control related to fruits and vegetables consumption (13, 15). Furthermore, educators were found perceiving school-based gardening as improving the dietary habits and social skills of students (16). Planting program could encourage adolescent students to participate in outdoor activities that could improve their academic achievements, social behaviors, and emotions (17). A previous study reported that better school performance was associated positively with happiness in high school students (18). Additional benefits from activities in green space include higher physical activity level (19), lower BMI, and lower prevalence rate of being overweight (20).

However, there were unclear areas that needed further study to examine the effects of school-based planting activities for mental well-being and dietary lifestyle behaviors of students. Although cross-sectional or ecological evidence suggested that greenness is protective against mental health issues, NCDs, and related deaths (19), there was limited evidence of causality between greenness exposure and these problems, particularly mental health, in the population (21). Moreover, alternative school-based planting activities and their effects required further investigation. For instance, a study reported that although adolescents (*N* = 30) consumed the recommended amount of fruits and vegetables after undertaking hydroponic gardening, they did not have their dietary habits changed significantly (22). Hydroponic planting is a system that grows crops such as leafy vegetables, herbs, and fruits with nutrient solutions in a controlled environment with equipment that could be vertically installed to efficiently utilize space (23). Other benefits include faster growth (24), higher productivity, and being less labor-intensive (23) than traditional planting. In the future study, a larger sample could be examined, and a low-cost hydroponic planting system of a smaller scale could be adopted.

Furthermore, the effectiveness of a health promotion program depends on more than a single modality. A review article of more than 30 studies pointed out that a multicomponent approach was considered the most effective way to promote healthy behavior among adolescents at school (25). Evans et al. (26) also concluded that multiple components should be included in behavioral change intervention for adolescents, and they found that students had better fruits and vegetables intake and related self-efficacy and knowledge, and weaker preference for unhealthy foods, after classroom activities, school gardening, and tasting foods than students who were involved in fewer components. In addition, interactive approach such as games in education was deemed motivational and effective to engage and support students in active learning (27).

## Study Aim

The study aimed to investigate the effects of school-based hydroponic planting integrated with health promotion activities in improving green space use, competence and satisfaction, healthy lifestyle, mental health, and health-related quality of life (QoL) among early adolescent students in secondary schools.

## Hypothesis

The hypotheses were (1) green space use and satisfaction would be higher in the intervention group of hydroponic planting than in the control group; (2) intervention group of hydroponic planting would have healthier lifestyle habits than the control group; (3) the intervention of integrating hydroponic planting and health promotion activities would significantly improve mental health and health-related QoL compared with the control group.

## Methods

### Design

The study design was a three-group comparison design (one control and two intervention groups).

### Eligibility Criteria

Inclusion criteria of participants were secondary 1–3 (grade 7–9) students; able to read and write Chinese; and not having an allergy to planting materials such as soil and plants. The inclusion criteria for schools were secondary schools, coeducation schools, and able to offer a venue for hydroponic planting. We neither assessed the mental health status of students nor required students to report their mental health diagnosis as our targeted variables were green space satisfaction and happiness among students who were functionally adaptive and eligible for education and activities in ordinary schools.

### Setting and Location of Data Collection

The students who participated in this project completed the questionnaires at school in Hong Kong.

### Interventions

School-based hydroponic planting and health promotion activity were the two intervention components in this project. One intervention group (A) participated in both components, while another group (B) joined only the health promotion activity. For group (A), they had undertaken hydroponic planting that integrated with at least one of the health promotion activities, such as, learning a balanced lifestyle, healthy eating, and physical exercise promotion. Both hydroponic planting and health promotion activities were implemented by the service-learning team of The Hong Kong Polytechnic University.

#### Design Basis

The intervention design was based on the potential benefits, contextual needs, and feasibility of hydroponic planting for secondary school students in Hong Kong. Literature indicated that natural green environments could help humans establish a more relaxing lifestyle and improve their states of mind, stress level, and physical activity. Numerous studies showed the evidence of positive mental health outcomes such as stress reduction and emotional stability associated with green space and green environments (28–38). Gardening is an effective mental health intervention for stress reduction (39); a study conducted by Hui (40) stated that visual or physical contact with the green space could improve both mental and psychological well-being in the Hong Kong context. The environment modification in the urban area to impact population health would be best achieved through local experimentation in health projects (41). School gardening is feasible to be integrated into the noncurriculum school events, under the aims of reinforcing social relationships and teamwork in student development as recommended by the Hong Kong Education Bureau (42). The hydroponic planting program aimed at impacting the awareness of green space and green eating of students (13–15, 26), as well as offering better options to overcome barriers related to school gardening participation to promote sustainable healthy lifestyles, and to inform worthwhile studies on the long-term goal of health outcomes in the future.

#### Equipment

The hydroponic planting systems were installed on the rooftop or in the garden of the participating schools before the project commenced. Other materials for health promotion activities were provided by the service-learning team.

#### Content

To run the hydroponic system, students needed to carry out procedures such as seed germination, seeding, transplantation, watering, and harvesting. The vegetables grew by students included Chinese cabbage and Chinese flowering cabbage. Regarding health promotion activities, each of them focused on one topic at a time, such as a balanced lifestyle, healthy eating, and physical exercise promotion. For instance, food labeling was taught in the balanced diet session, and simple stretching exercise was demonstrated in the physical exercise promotion session.

#### Procedure

Students had to visit and maintain the hydroponic planting system two times a week for an hour to perform the planting tasks. On the other hand, they had to join health promotion activities weekly, which took around 1 h at lunchtime or after school. The session included a 30-min talk about the topic of concern, followed by a 30-min game to reinforce the key messages delivered.

#### Schedule

The period for hydroponic planting began in October 2016 and ended in May 2017. The health promotion activities were held between March and April 2017 for 6 weeks.

#### Standardization

The training provided by the service-learning team was standardized across schools in terms of hydroponic planting system, planting technique, plants to grow, and teaching materials for health promotion activities such as posters and leaflets. However, each intervention group student was free to participate in any topic of health promotion activities over the project period.

### Control Group

The control group participated in neither hydroponic planting nor health promotion activities. They had undergone their usual school activities and left after school.

### Outcome Assessment

#### Primary Outcomes

Green space use and satisfaction was assessed in three domains, they were (1) green space distance and use (three items) (2, 30, 43–45) green space activity and competence (six items) (46, 47); and (3) green space sense and satisfaction (five items) (48). Each item was measured with an ordinal scale. A higher score indicates more frequent green space use in the past 4 weeks or the level of agreement. The content validity was examined by a secondary school teacher, and two health researchers at The Hong Kong Polytechnic University. The item relevancies in terms of item-content validity index (CVI) were 100%, which supported the content validity of the instrument (49). The internal consistency of the instrument was satisfactory (Cronbach's alpha = 0.896), which was above 0.8 (50).

#### Secondary Outcomes

The secondary outcomes included life happiness, resistance to substance use, dietary habits, hand hygiene, emotions and friendship, physical activity, depressive symptoms, and health-related QoL.

Life happiness was measured by the Delighted-Terrible Faces (DT-Faces) Scale, which was a 7-point ordinal visual analog scale (VAS) (51, 52). The score ranged from 1 = most unhappy (face G) to 7 = most happy (face A).

The items of resistance to substance use, dietary habit, hand hygiene, emotional feelings and friendship, and physical activity were adopted from the Global school-based Student Health Survey (GSHS) developed by the WHO (53), which was used to assess the behavioral risk factors and protective factors in adolescents aged between 13 and 17. The GSHS had been used in Hong Kong among secondary school students (54).

The resistance to substance use was assessed on a 4-point Likert scale where a higher score shows a higher level of agreement with resisting temptation from peers about substance use to relieve stress.

The dietary habit was measured by four ordinal scales that quantified the frequencies of having breakfast and eating fruits, vegetables, or drinking carbonated soft drinks per day in the last 30 days. A higher score indicates a more healthy diet.

Hand hygiene was assessed with five ordinal scales about the frequencies of washing hands before eating, after the toilet, at usual times or at school time, and using soap or sanitizer for handwashing in the last 30 days. A higher score implies more often handwashing.

Emotional feelings and friendship were measured with ordinal scales and dichotomous scale regarding how often the student felt lonely, could not sleep due to worries, or whether the daily activities were hindered by sadness or desperation for 2 or more weeks in the last 12 months; and how many close friends the student had. A higher score indicates better mental health.

Physical activity was assessed with five ordinal scales where (1) the number of days of being physically active for at least 60 min per day or per week; (2) the number of days having physical education (PE) lessons per week; (3) the number of sports teams joined annually; and (4) the number of hours sitting per day, were measured. The higher score is associated with a more physically active lifestyle.

Depressive symptoms in the last 2 weeks were assessed by using the modified Patient Health Questionnaire-9 (PHQ-9). The two items of PHQ-9 “Moving or speaking so slowly that other people could have noticed? Or the opposite—being so fidgety or restless that you have been moving around a lot more than usual,” and “Thoughts that you would be better off dead or of hurting yourself in some way” were excluded because they were not considered related to the study aim. The instrument had satisfactory internal consistency (Cronbach's alpha = 0.84) and good test–retest reliability (intraclass correlation coefficient = 0.80) (55) in Chinese adolescents. The previous study supported its measurement invariances by age and gender (56). A higher score shows a higher level of depressive moods.

Health-related QoL was measured with a modified 12-item Short Form Survey (SF-12). Items 4–7 and items 9–11 were assessed by 5-point ordinal scales in the modified instrument instead of dichotomous scales and 6-point ordinal scales in the original version (57). Although the mental health subscale had lower internal consistency (Cronbach's alpha = 0.34) in a previous study (58), a recent study reported that the performance of measuring the two main components (physical and mental) in SF-12 appeared to be comparable to SF-36 among Chinese adolescents (59). A higher score indicates poorer health-related QoL.

#### Demographic Characteristics

Demographic variables such as gender, age, school grade, years lived in Hong Kong, cohabitants, religion, and the reception of students of mental health services were assessed in the questionnaire. Age and years lived in Hong Kong were continuous variables, the others were categorical.

#### Procedure

In June 2017, after the intervention period, the service-learning team distributed the questionnaires to students in the three groups to measure the outcome variables. The assessment was not done immediately after intervention in May because of the schedule of school to complete the assessment after the school exam period.

### Sample Size and Group Allocation

The sample size was not predetermined as two schools were conveniently recruited. There were 18 districts and 506 secondary schools in Hong Kong between 2016 and 2017 (60). All Form 1–3 (grade 7–9) students were principally allowed to participate in the intervention activities but only those who were available based on their school schedule and routine could join. Those who did not join any intervention components were labeled as the control group given the consents available.

### Blinding

The students and parents received information sheets and provided informed consent to participate in the project. However, they were blinded to the study hypothesis.

### Statistical Methods

#### Demographic Characteristics

Categorical variables of demographic characteristics between groups were compared by using Pearson's chi-squared test with simulated *p*-value (based on 2,000 replicates) (61). If for a variable, there were more than 20% of cells with the expected count of <5 (62), additionally a Fisher's exact test was run with Monte Carlo simulation based on 2,000 replicates (63). Continuous variables between groups were compared by using the Welch's ANOVA test (64).

#### Primary and Secondary Outcomes

Total scores of subscales of green space use and satisfaction, and subscales of GSHS, as well as total scores of PHQ-9 and SF-12, were computed, respectively. Along with the life happiness score, all these scores between groups were compared by using the Welch's ANOVA test. Partial eta-squared and its 95% CI were also computed (65). The cutoffs of eta-squared are 0.01, 0.06, and 0.14 to indicate small, medium, and large effect sizes (66). In addition, generalized estimating equations (GEE) (67) were used to compute the parameters (regression coefficients and standard errors) of primary outcomes and secondary outcomes, which regressed on the group, sex, and school grade. The error distribution was assumed following the Gaussian family. The Little's missing completely at random (MCAR) test (68) was nonsignificant (*p* = 1.00), which indicates that the missing data were completely at random, therefore no imputation was done. The significance level was set at 0.05. All analyses were done on R 4.0.3 (69) and RStudio 1.4.1103 (70).

## Results

### Participant Flow

We invited 576 students and none of them were excluded based on recruitment criteria; however, 23 of them did not consent to participate. In one school, we had recruited 240 students and, in another school, we recruited 313 students. There were 123 students who had participated in both intervention components; 235 students who only joined health promotion activities; and 195 students who were labeled as control group participants. Finally, 553 cases were analyzed. The recruitment period was September 2016. The intervention period began in October 2016 and ended in May 2017. The data were then collected in June 2017.

### Demographic Data

There were significant differences in the ratio of control to intervention group students between schools (Table 1). There were only 25% of the students in the first school who were control group participants, but there were 43% in the second school (*p* < 0.001). There were more senior students in the control group and Group A, but more junior students in Group B (*p* = 0.0025). The gender imbalance was also observed across groups, but it was more evident in the control group which had over three times more males than females (*p* = 0.02).

**Table 1:** Demographic characteristics.

		**Control**	**Group A**	**Group B**	**χ** ^**2**^	***p* (Fisher *p*)**
School	1	59	68	113	22.89	0.00050
	2	136	55	122		
Form	1	53	37	90	16.79	0.0025
	2	72	31	85		
	3	70	54	60		
Sex	M	151	78	172	7.61	0.020
	F	44	45	62		
Live with father	No	32	23	49	1.32	0.50
	Yes	162	100	186		
Live with mother	No	19	13	26	0.18	0.93
	Yes	175	110	209		
No. of siblings	0	64	35	73	9.33	0.51 (0.61)
	1	72	58	105		
	2	30	20	29		
	3	3	5	8		
	4	2	1	3		
	5	2	0	0		
Live with paternal grandfather	No	180	119	221	2.19	0.35
	Yes	14	4	14		
Live with paternal grandmother	No	168	105	212	2.23	0.35
	Yes	26	18	23		
Live with maternal grandfather	No	191	120	233	1.45	0.48 (0.46)
	Yes	3	3	2		
Live with maternal grandmother	No	189	119	227	0.73	0.77
	Yes	4	4	8		
Live with others	No	188	116	224	0.80	0.67
	Yes	7	7	11		
Religion	No	155	87	167	20.19	0.012 (0.051)
	Buddhism	15	13	19		
	Catholics	2	1	3		
	Protestant	20	18	45		
	Taoism	1	0	0		
	Others	0	3	0		
Mental health service	Never	65	60	82	7.29	0.51 (0.60)
	Teacher, social worker, and nurse	10	11	13		
	NGO	17	12	23		
	Doctor without medication	10	5	8		
	Doctor with medication	5	1	1		
		**Mean (SD)**	**Mean (SD)**	**Mean (SD)**	***F***	***p***
Age (*N* = 551)		14.1 (1.19)	14.1 (1.17)	13.8 (1.12)	3.71	0.026
Years lived in HK (*N* = 534)		10.7 (4.88)	10.5 (5.08)	10.4 (5.03)	0.15	0.86

There were no significant differences in cohabitants among the students. Around 80 and 90% of the students were living with their father and mother, respectively. Nearly one-third of the students did not have siblings. Over 60% of them had one to two siblings. Around 12% of the students lived with paternal grandmother, and <6% lived with paternal grandfather. Moreover, <3% of the students lived with maternal grandparents.

Over 80% of the students did not have a religious belief in the control group, compared with the 70% in the intervention groups (Fisher's exact test, *p* = 0.051). For those who had religious beliefs, Buddhism (9%) and Protestantism (15%) were the major religions. On the other hand, a majority of the students (64%) claimed that they did not receive any mental health services. Less than 10% of the students reportedly required follow-up by the physician. Overall, the mean age of the students was 14. The differences between groups were small though the test was statistically significant (*p* = 0.026). On average, the number of years lived in Hong Kong was around 10.5 years among the students.

### ANOVA

As for the primary outcomes, Group A had the highest score in “Green space distance and use” (*p* < 0.001) and “Green space activity and competence” (*p* < 0.001) (Table 2). The control group had the lowest scores in these domains. The effect sizes were moderate to large. Both Group A and B scored higher than the control group in “Green space sense and satisfaction” (*p* = 0.024) and life happiness (*p* = 0.0061), but the effect sizes were however smaller. The dietary habit score in Group A was the highest among all groups (*p* = 0.021), while the score in the control group was the lowest. The effect size of the between-group difference was moderately small. Although the result of “resistance to substance use” was not significant, the scores in Group A and Group B were higher than the control group and the effect size was moderate to small. However, we did not find significant differences in hand hygiene, emotions and friendship, physical activity, depressive symptoms, and health-related QoL between groups after the intervention period.

**Table 2:** Between group comparisons for primary and secondary outcomes.

	***N***	**Range**	**Control**	**SD**	**Group A**	**SD**	**Group B**	**SD**	***F***	***p***	**η** ^**2**^	**95%**	**CI**
			**Mean**		**Mean**		**Mean**					**Lower**	**Upper**
Green space distance and use	550	0–9	1.07	1.51	2.05	1.82	1.64	1.65	14.32	<0.001	0.09	0.03	0.15
Green space activity and competence	549	6–30	16.4	5.65	21.9	5.33	17.7	5.33	40.81	<0.001	0.21	0.13	0.28
Green space sense and satisfaction	544	5–20	13.9	3.39	14.6	2.86	14.7	2.82	3.79	0.024	0.02	0.00	0.06
Life happiness	553	1–7	4.85	1.23	5.11	1.17	5.21	1.12	5.19	0.0061	0.03	0.00	0.08
Resistance to substance use	281	1–4	2.36	1.57	2.85	1.32	2.56	1.56	2.24	0.11	0.03	0.00	0.09
Dietary habits	542	0–22	11.4	3.00	12.3	3.27	12.1	3.40	3.91	0.021	0.03	0.00	0.07
Hand hygiene	541	0–20	14.7	3.56	14.9	3.72	15.0	3.49	0.25	0.78	0.00168	0.00	0.02
Emotions and friendship	539	0–12	8.69	2.66	8.31	2.57	8.6	2.47	0.81	0.45	0.00537	0.00	0.03
Physical activity	511	1–28	9.69	5.54	9.96	4.32	10.5	5.16	1.27	0.28	0.00819	0.00	0.03
Depressive symptoms	522	0–21	15.6	4.94	16.2	4.48	15.9	4.57	0.46	0.63	0.00310	0.00	0.02
Health related quality of life	467	1–45	27.5	5.40	27.4	5.00	27.1	5.89	0.31	0.73	0.00232	0.00	0.02

*F, F-value of ANOVA. η^2^, eta-squared*.

### Regression

After adjusting for gender and school grade, the scores in “green space distance and use” (*p* < 0.001) and “green space activity and competence” (*p* < 0.05) were significantly higher in the intervention groups than in the control group, particularly in Group A (Table 3). Group A was also associated with a higher score in the resistance to substance use (*p* < 0.01) and dietary habits (*p* < 0.05) when compared with the control group. Interestingly, Group B had a higher score in “green space sense and satisfaction” (*p* < 0.05) and life happiness (*p* < 0.01) when compared with the control group. Concerning gender difference, females had higher scores in “green space activity and competence” (*p* < 0.05), “green space sense and satisfaction” (*p* < 0.05), and dietary habits (*p* < 0.01). However, females appeared to be less physically active (*p* < 0.001) and had worse perceived health-related QoL (*p* < 0.05). Regarding school grade difference, Form 3 (grade 9) students had lower score in “green space sense and satisfaction” (*p* < 0.05) and physical activity (*p* < 0.01) but less depressive score than Form 1 students (*p* < 0.05). Nonetheless, Form 2–3 students had lower resistance to substance use (*p* < 0.001) and worse health-related QoL than Form 1 students (*p* < 0.01).

**Table 3:** Primary and secondary outcomes regressed on group, sex, and school grade.

	**(Intercept)**	**Group A**	**Group B**	**Female**	**Form 2**	**Form 3**
	**B (SE)**	**B (SE)**	**B (SE)**	**B (SE)**	**B (SE)**	**B (SE)**
Green space distance and use	1.08 (0.15)^***^	1.02 (0.2)^***^	0.57 (0.15)^***^	−0.09 (0.15)	0.11 (0.18)	−0.09 (0.16)
Green space activity and competence	16.33 (0.52)^***^	5.39 (0.64)^***^	1.2 (0.53)^*^	0.98 (0.49)^*^	−0.16 (0.56)	−0.3 (0.56)
Green space sense and satisfaction	13.97 (0.31)^***^	0.66 (0.36)	0.75 (0.31)^*^	0.54 (0.26)^*^	0.03 (0.33)	−0.61 (0.3)^*^
Life happiness	4.88 (0.12)^***^	0.27 (0.14)	0.34 (0.11)^**^	0.08 (0.11)	0.001 (0.13)	−0.14 (0.12)
Resistance to substance use	2.96 (0.22)^***^	0.62 (0.24)^**^	0.07 (0.22)	0.06 (0.18)	−0.71 (0.2)^***^	−0.95 (0.22)^***^
Dietary habits	11.49 (0.31)^***^	0.85 (0.38)^*^	0.57 (0.31)	0.87 (0.31)^**^	−0.2 (0.35)	−0.64 (0.35)
Hand hygiene	14.83 (0.35)^***^	0.16 (0.44)	0.17 (0.35)	0.61 (0.32)	−0.16 (0.39)	−0.46 (0.37)
Emotions and friendship	9.04 (0.26)^***^	−0.32 (0.31)	−0.11 (0.25)	−0.42 (0.25)	−0.37 (0.27)	−0.34 (0.27)
Physical activity	11.06 (0.56)^***^	0.53 (0.58)	0.75 (0.54)	−1.86 (0.44)^***^	−1.01 (0.55)	−1.59 (0.57)^**^
Depressive symptoms	16.35 (0.44)^***^	0.6 (0.55)	0.11 (0.47)	−0.28 (0.45)	−0.58 (0.48)	−1.17 (0.49)^*^
Health related quality of life	25.87 (0.61)^***^	−0.22 (0.64)	−0.23 (0.58)	1.13 (0.54)^*^	1.82 (0.63)^**^	2.07 (0.63)^***^

*B, regression coefficient of generalized estimating equations (GEE) model*.

* *p < 0.05*,

** *p < 0.01, and*

*** *p < 0.001*.

## Discussions

### Limitations

First, the representativeness of the sample might be undermined because of convenience sampling. Second, the number of schools was only two and might not reflect the characteristics of the larger school population. Third, the sample size of students was not predetermined and an intervention study with a large sample could be difficult to manage. Four, in the results, the percentage of missing data was high (49%) for the item asking about resistance to substance use if invited by peers. The hypothetical question could be modified to measure actual invitations and responses.

The limited school space also restricted the number of planting sets to be installed and the number of student participants. Moreover, the hydroponic planting took 8 months, two times per week; but the weekly health promotion activities only took 6 weeks. The comparison between intervention groups could be difficult. Further, though the control group participated in neither intervention group, the participants were living in communities that were subject to confounding effects.

There was no baseline assessment as the project activities could only begin according to the school schedule in the academic year. In addition, there was no random allocation as students in each school participated in either intervention group or control group according to their school schedule and availability, and they were free to join any of the health promotion activities within intervention period. Since there were only two schools and students who participated in the project based on their availability, we did not implement cluster randomization. Hence, there could be a risk of effects contamination across groups within a school.

The construct validity and test–retest reliability of the green space assessment scale require further study evidence. Also, a previous study showed that GSHS only had moderate internal consistency and test-retest reliability (71), so further study is needed. Furthermore, the study instrument was a self-administered questionnaire, there could be recall bias in the answers. Moreover, the data were collected 1 month after the completion of the intervention, which might also introduce bias in the responses.

### Generalizability

Although the generalizability of the study results might be lowered by the limitations identified, the interventions are considered feasible and could be applied among early adolescent students in secondary schools in Hong Kong and even the urban areas in other cities to improve green space use and satisfaction, dietary lifestyle, and resistance against peer pressure of substance use or other risky behaviors of students.

### Interpretations

The hypothesis that green space use and satisfaction would be higher in the intervention group of hydroponic planting was partially supported. The intervention group integrated with hydroponic planting had better green space use and competence in green activities than the control group. On the other hand, interesting findings were that students who had undertaken only health promotion activities had their green space satisfaction and life happiness better than the control group even after sex and school grades were adjusted for. These results implied that the subjective appraisal of the physical environment and happiness in life could be promoted by general health promotion activities but not exclusively by planting activities. A previous study reported a significant and moderate to the strong association between happiness and psychological well-being among high school students (18). Based on our results, both health promotion activity and hydroponic planting are feasible to support the mental well-being of students.

The hypothesis that the intervention group of hydroponic planting would have healthier lifestyle habits was partially supported. Hydroponic planting integrated with health promotion activities was associated with better dietary habits and resistance to substance use than the control group. However, the results were not significant for other lifestyle domains in GSHS. The results in this study provide new evidence to support the effectiveness of hydroponic planting in improving dietary habits that were not found by Anderson and Swafford (22). Nonetheless, we did not find a significant difference in physical activity between groups, which was a finding different from the conclusion made by James et al. (19) that green space was associated with higher physical activity levels. However, our project focused on planting in a hydroponic system but not expanding the green area of the school to be sufficient for physical activity to which James et al. (19) referred.

In addition, the findings that deserve attention are that females had better green space competence and satisfaction than males, and senior students had worse results than junior students in these respects. Senior students had lower resistance to substance use than junior students. Moreover, females also had better dietary habits than males. However, it was unclear whether Hong Kong females would have a lower or higher risk for stress-related eating as found by Jääskeläinen et al. (5). We also did not find significant differences in depressive symptoms, emotions, and life happiness between sexes in adjusted analysis. Based on these results, senior males could be the targeted group of students in need in terms of hydroponic planting integrated with health promotion activities to improve their green space use and competence, dietary habits, and resistance to substance use.

The hypothesis that the intervention of integrating hydroponic planting and health promotion activities would improve mental health (including emotions and depressive symptoms) and health-related QoL was not supported as the results were nonsignificant. We did not have strong evidence to support the causality between green space activities and mental health, neither did James et al. (19) nor Gascon et al. (21). However, future studies could address the study limitations and further examine the associations between school-based hydroponic planting and the targeted outcomes in adolescents. In addition, practical and cultural hindrances should be overcome to maintain the sustainability of the school-based planting activities (72).

Government and organizational health planners and policymakers should recognize the benefits and positive impacts on lifestyle behaviors and psychological well-being of adolescent students through participation in hydroponic planting integrated with school-based health promotion activities as noncurricular activities. Governments should identify green space activities as one of the core school activities to nurture a sense of the health of students in the natural environment, and draft clear policies to direct the translation from an over-arching goal to feasible actions at the school level (22). Health planners in the government and educational agencies should design school-based green area activities with greater granularity and better organization, so that health promotion activities could be fitted to the school schedule and routine and be effective and also sustainable (73). Based on our results, we offered the insights of designing several key areas for promoting the health of students in green space as an initiative of health-promoting schools, which has been advocated by the school health policy of government locally (74). The school health policy of establishing a healthy school environment should include a clear delineation of the role of a school to provide a health-promoting learning environment for students (75). Our findings give a new direction to generate insight from the knowledge gap filled, for the references of schools to design the appropriate program to satisfy the aim of promoting wellness of students concerning both lifestyle behaviors and psychological health and relevant factors. In a future study, random sampling could be implemented with larger sample size. Missing data problems could be minimized by better design of the questionnaire. The intervention period and duration shall be the same across groups, with both baseline and posttest measurements. Posttest should be done right after the intervention period. RCT should fit the school schedule to generate new evidence for adolescent health in the school communities.

## Conclusions

Our study shows that the school-based hydroponic planting integrated with health promotion activities was feasible, and generally, better than health promotion activity alone, to improve green space use and competence, dietary habits, and resistance to substance use among early adolescent students in secondary schools in urban areas. This intervention program could be introduced to schools in different geographical, socioeconomical, and cultural contexts to further examine its effects and generalizability. Based on our results, the importance of the association among green space use, competence and satisfaction, and healthy lifestyle should not be overlooked to promote adolescent health at school. Future studies should address the limitations identified, for example, designing RCT that could fit school schedules to generate new evidence for adolescent health in the communities.

## Data Availability Statement

The raw data supporting the conclusions of this article will be made available by the authors, without undue reservation.

## Ethics Statement

The studies involving human participants were reviewed and approved by Human Subjects Ethics Sub-Committee of The Hong Kong Polytechnic University (ID: HSEARS20160618002). Written informed consent to participate in this study was provided by the participants' legal guardian/next of kin.

## Author Contributions

HT, CH, KL, YL, KL, CKCW, and CSTW: conceptualization, methodology, validation, investigation, project administration, and resources. HT, CH, KL, YL, KL, CKCW, CSTW, and SK: formal analysis, writing-original draft preparation, and writing-review and editing. CSTW: supervision and funding acquisition. All the authors have read and approved the final version of the manuscript.

## Funding

This research was funded by Lee Hysan Foundation under the Service Learning Project in Collaborative Care in School Health and Safety (SN2S01) and the Service Learning Fund of the Hong Kong Polytechnic University. The funding body has no role in the design of the study and collection, analysis, and interpretation of data and in writing the manuscript.

## Conflict of Interest

The authors declare that the research was conducted in the absence of any commercial or financial relationships that could be construed as a potential conflict of interest.

## Publisher's Note

All claims expressed in this article are solely those of the authors and do not necessarily represent those of their affiliated organizations, or those of the publisher, the editors and the reviewers. Any product that may be evaluated in this article, or claim that may be made by its manufacturer, is not guaranteed or endorsed by the publisher.
